# Propulsive efficiency of frog swimming with different feet and swimming patterns

**DOI:** 10.1242/bio.022913

**Published:** 2017-03-16

**Authors:** Fan Jizhuang, Zhang Wei, Yuan Bowen, Liu Gangfeng

**Affiliations:** State Key Laboratory of Robotics and System, Harbin Institute of Technology, Harbin 150080, China

**Keywords:** Frog, Swimming patterns, Foot shapes, Hydrodynamics, Propulsive efficiency

## Abstract

Aquatic and terrestrial animals have different swimming performances and mechanical efficiencies based on their different swimming methods. To explore propulsion in swimming frogs, this study calculated mechanical efficiencies based on data describing aquatic and terrestrial webbed-foot shapes and swimming patterns. First, a simplified frog model and dynamic equation were established, and hydrodynamic forces on the foot were computed according to computational fluid dynamic calculations. Then, a two-link mechanism was used to stand in for the diverse and complicated hind legs found in different frog species, in order to simplify the input work calculation. Joint torques were derived based on the virtual work principle to compute the efficiency of foot propulsion. Finally, two feet and swimming patterns were combined to compute propulsive efficiency. The aquatic frog demonstrated a propulsive efficiency (43.11%) between those of drag-based and lift-based propulsions, while the terrestrial frog efficiency (29.58%) fell within the range of drag-based propulsion. The results illustrate the main factor of swimming patterns for swimming performance and efficiency.

## Abstract (precis)

**Summary:** To explore propulsion in swimming frogs, this study analyzed mechanical efficiencies based on data describing aquatic and terrestrial feet and swimming patterns.

## INTRODUCTION

The bodies of aquatic and terrestrial animals have diverse shapes and moving patterns that result in distinct swimming performances. Fish generally wave their body-caudal fins (BCF) and median or paired fins (MPF) for propulsion through water (Sfak et al., 1999; Chu et al., 2012); these fins create minimal drag and strong lift-based thrusts, leading to swimming efficiency as high as 80% (Fish, 1996). In contrast, drag-based propulsions like paddling and flapping are utilized by terrestrial mammals or semi-aquatic animals, which therefore have low swimming efficiencies. For example, turtles and crabs swim with their flapping foils (Roper et al., 2011; Kim et al., 2013), and frogs swim using their webbed feet (Nauwelaerts et al., 2005). Squid and jellyfish propel themselves through water by employing variable cavities (Shvalb et al., 2012; Rahman el al., 2013).

Research into frogs' swimming methods treats the webbed foot as the main propulsive unit, and the foot shape and motion patterns differ by species. Different methods can be used to analyze propulsive forces related to morphological and kinematic patterns (Richards and Clemente, 2013). Richards explored the hydrodynamic force in swimming frogs through a modified blade element approach (Richards, 2008), analyzing the thrust contribution from translational and rotational motions of the hind legs and concluding that aquatic and terrestrial frogs have distinct kinematic patterns. Some terrestrial frogs exhibited higher translational thrust than rotational thrust, while rotational thrust was higher in some aquatic frogs (Richards, 2010).

The propulsion mechanism is a key factor in swimming performance and efficiency. Drag-based thrust can only be generated when the velocity of the webbed foot is opposite to the direction of swimming. Therefore, as the body moves forward, the foot cannot generate drag-based thrust, while lift-based thrust continues to work through rotation. However, only a few studies have explored swimming propulsion mechanisms.

Johansson and Lauder (2004) observed frogs swimming and recorded flow field data through digital particle image velocimetry (DPIV) to determine if any aspect of lift-based propulsion is characterized by a U-shaped vertex like that observed in foot-propelled swimming birds (Johansson and Norberg, 2003). Sandra Nauwelaerts et al. (Nauwelaerts et al., 2005; Stamhuis and Nauwelaerts, 2005) researched the propulsive force and compared jumping and swimming thrust in frog via DPIV. They found the impulses of swimming and jumping differ, and some hypotheses regarding to swimming performances were tested based on their momentum–impulse approach. The results of our previous work (Zhang et al., 2014; Fan et al., 2014) suggest that some kinds of aquatic frogs may utilize lift-based propulsion.

Swimming animals, especially terrestrial ones, evolved such that improved swimming performance developed at the expense of terrestrial locomotion. Therefore, the comparison of swimming performances among different frog species yields interesting findings, because they move both on land and in the water. Previous works on frog swimming mechanism analysis lack the study on influence between lift propulsion and swimming efficiency; therefore, this paper analyzes the frog swimming performances and mechanical efficiencies based on computational fluid dynamic (CFD) simulations.

## RESULTS

### Swimming performance

As previously introduced, the motions were divided into ankle trajectories, ankle accelerations at the trajectories, and the webbed foot's angular accelerations (Zhang et al., 2015). As ankle movement was analyzed separately, ankle track emerged as one of the most obvious indicators of different swimming patterns, demanding that more attention be paid to the magnitude of acceleration in order to evaluate the foot's translational component resulting from the hind leg's movements. With the simulation method, swimming results computed from combination of different swimming patterns and foot models could present the detailed illustrations which cannot extract from direct animal observations.

**Fig. 1.**
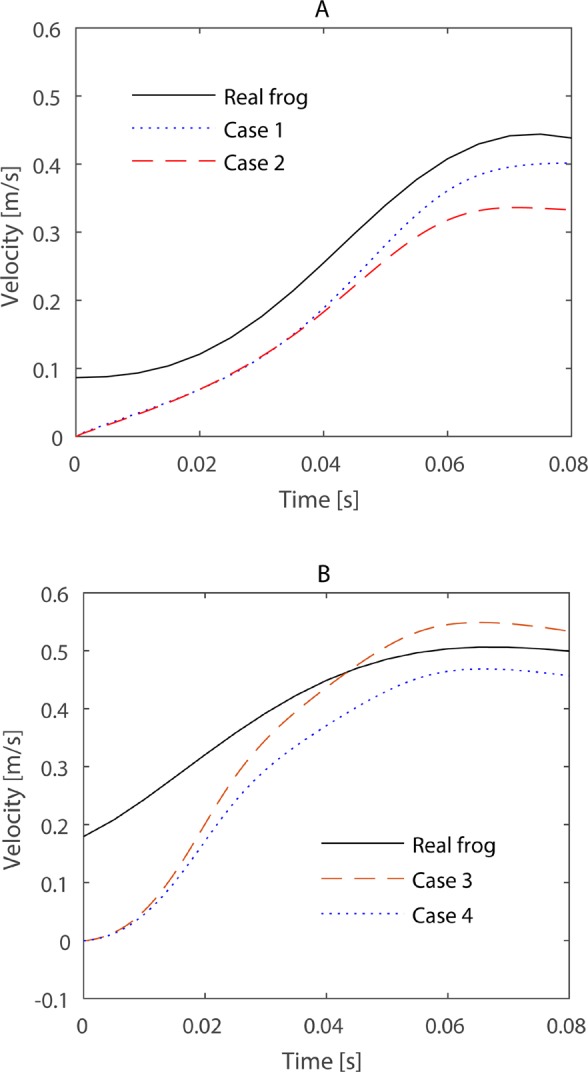
**Results of body velocities.** The results regarding terrestrial (A) and the aquatic (B) swimming patterns. The curves noted as real frog means the observed data from the two frogs' swimming trial as in Figs 6 and 7. The curves noted as cases 1 to 4 are results from the CFD simulations.

**Fig. 2.**
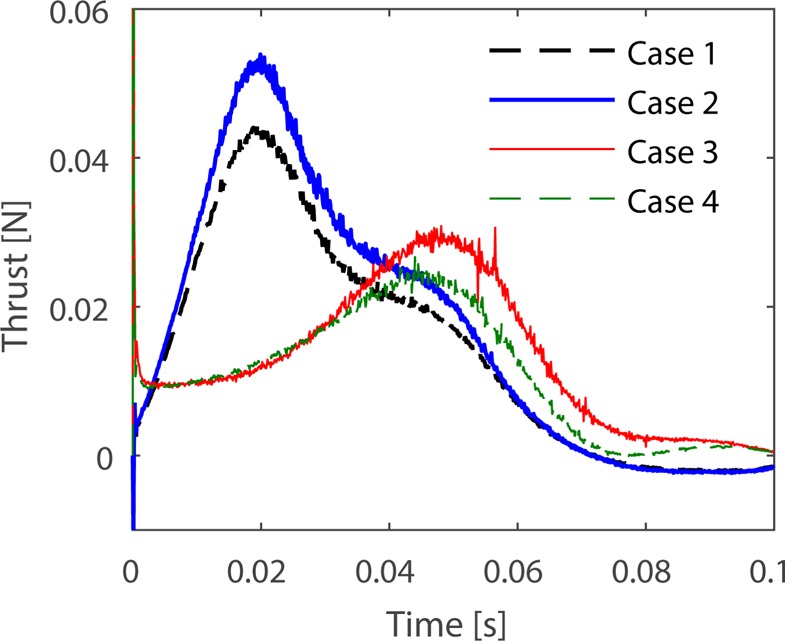
**Results of hydrodynamic thrust on feet.** Cases 1 to 4 are results from the CFD simulations previously described.

The frogs' swimming velocities were calculated to evaluate their performances, and the results are shown in Fig. 1. The simulation results from the terrestrial foot model showed higher swimming velocities in both swimming patterns, consistent with the results from Richards and Clemente (2012). Fig. 2 shows the foot thrust computed from CFD simulations, and case 1 and 2 results from the terrestrial swimming pattern, while case 3 and 4 results from aquatic swimming patterns. The swimming patterns here determined the propulsive performance, since the foot aspect ratio (AR) variation altered slightly in thrust magnitude and maintained the same power peak during propulsion. The aquatic webbed foot area was equal to the terrestrial's, and intense aquatic swimming pattern resulted in the fastest swimming. However, it is necessary to evaluate whether the raised performance is efficient.

### Propulsive efficiencies

With the data collected in Fig. 2 and the joint torques computed by Eqn (4), the swimming efficiency during propulsive phase could be obtained by Eqn (5). In Table 1, the swimming patterns performed slightly better efficiency with right-paired webbed feet, though in both swimming patterns terrestrial feet resulted in higher input and output works. In case 1, changing the terrestrial feet into large feet with a constant AR resulted in more input work and faster swimming, and the efficiency of terrestrial swimming reached 0.3526. In case 2, though the efficiency slightly decreased, the input work dropped sharply by 27%, thus requiring lower muscle output. The aquatic swimming pattern with terrestrial feet in case 3 resulted in a little efficiency change, but consumed more energy relative to that in case 4. Though the two foot shapes assessed in this paper made little difference in combination with the same frog swimming pattern, the right feet improved the efficiency slightly.

**Table 1.**
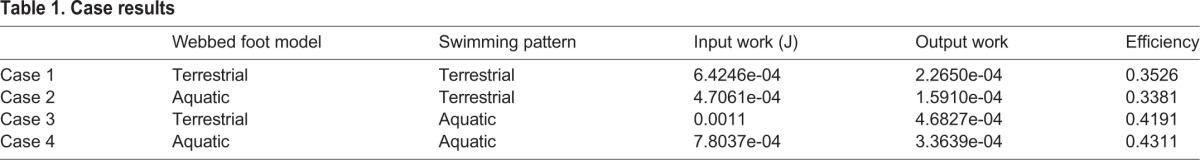
**Case results**

The CFD simulation with the real terrestrial frog data (that is, the terrestrial foot model before lengthening) was also calculated to make a contrast between real frog swimming trails. The foot parameters are as follows: *a*=1.5 mm, *R*=19.6 mm, *α*=18°, AR=1.27, area=113.2 mm^2^. According to the CFD results, the propulsive efficiency or mechanical efficiency of the real terrestrial frog was 0.2958. To further analyze the results that the aquatic swimming here is more efficient, hydrodynamic analysis should be conducted.

### Hydrodynamic results

**Fig. 3.**
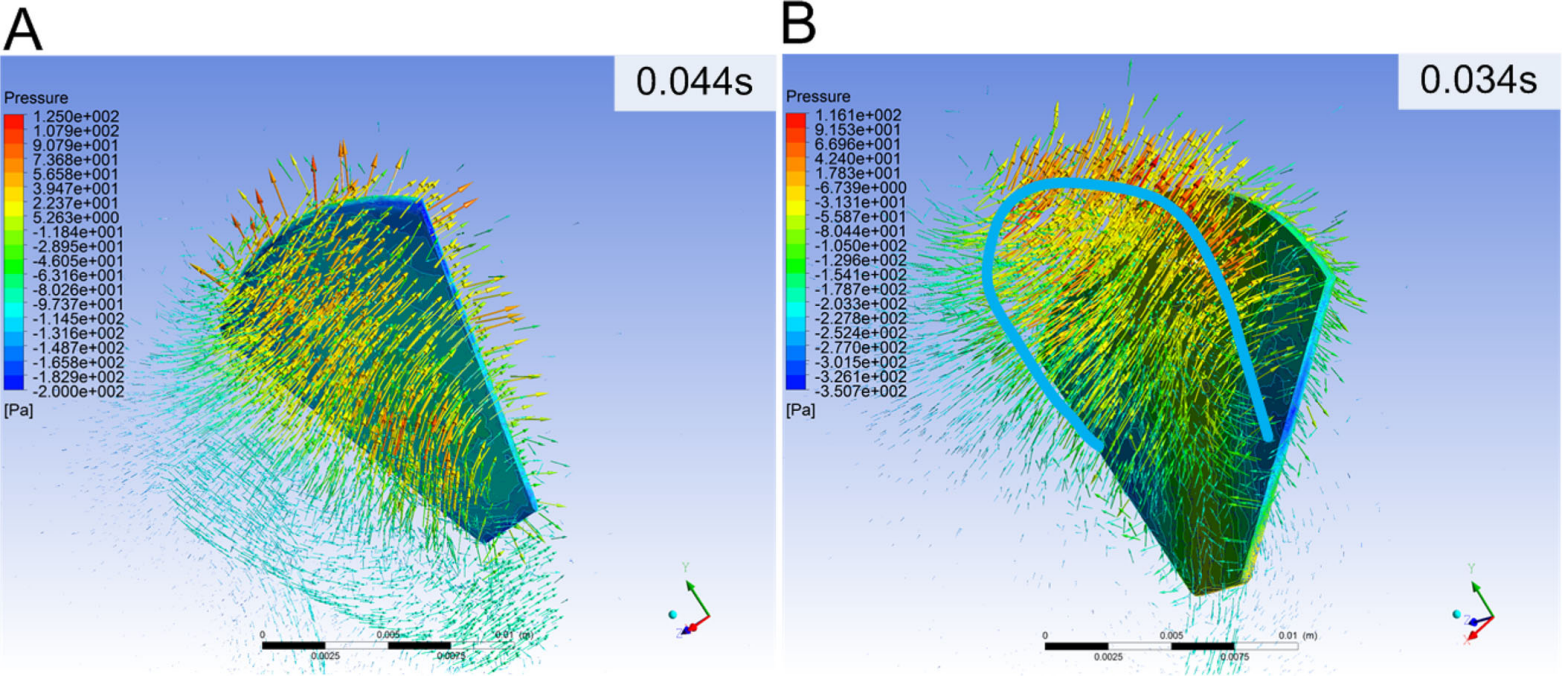
**Flow structure around the feet.** The arrows represent the velocity vector in flow field and pressure distribution is color mapped on the surfaces. (A) The fluid velocity and pressure distribution on the suction face in a terrestrial swimming trial at the time of 0.044 s. (B) The aquatic swimming trial at the time of 0.034 s, and two low pressure cores formed on the middle sides. The U-shaped vortex is noted as a bold blue line.

To analyze the influence between propulsive mechanism and swimming efficiency caused by different swimming patterns and webbed-foot shapes, hydrodynamic data from the CFD simulations were extracted. Fig. 3 shows the flow structure around frog feet.

Both kinds of frogs produced apparently different flow structures. For terrestrial swimming, the webbed foot rotated at a low speed at 0.044 s when the foot decelerated but angularly accelerated to push the added mass fluid backwards, so the velocity distributed uniformly above the suction face towards distal edge (Fig. 3A). For aquatic swimming, it intensely accelerated both by translation and rotation at the beginning of swimming, and with the foot rotation the fluid formed a U-shaped vortex (Fig. 3B) as described in the diving bird (Johansson and Norberg, 2003) which referred to a sign of lift-based propulsion. The U-shaped vortex is noted as a bold blue line in Fig. 3. The terrestrial frog showed no lift feature, while the aquatic frog generated a lift thrust in the second half of the propulsion. That is considered to be the reason for the higher efficiency in aquatic swimming.

For the influence caused by the webbed foot shapes, the hydrodynamic forces exerted on the foot were analyzed for pressure distribution. The foot's hydrodynamic pressure center was extracted from the CFD results. The hydrodynamic center in the terrestrial foot model was identified as 2 mm longer than the center in the aquatic foot, because the terrestrial feet were created by lengthening the foot to maintain the same area as the aquatic feet. A hydrodynamic pressure center closer to the outside of the foot would cause higher torques in the joints. The distal point on the foot achieved a greater velocity when the ankle rotated, which also resulted in the larger thrust shown in Fig. 2.

**Fig. 4.**
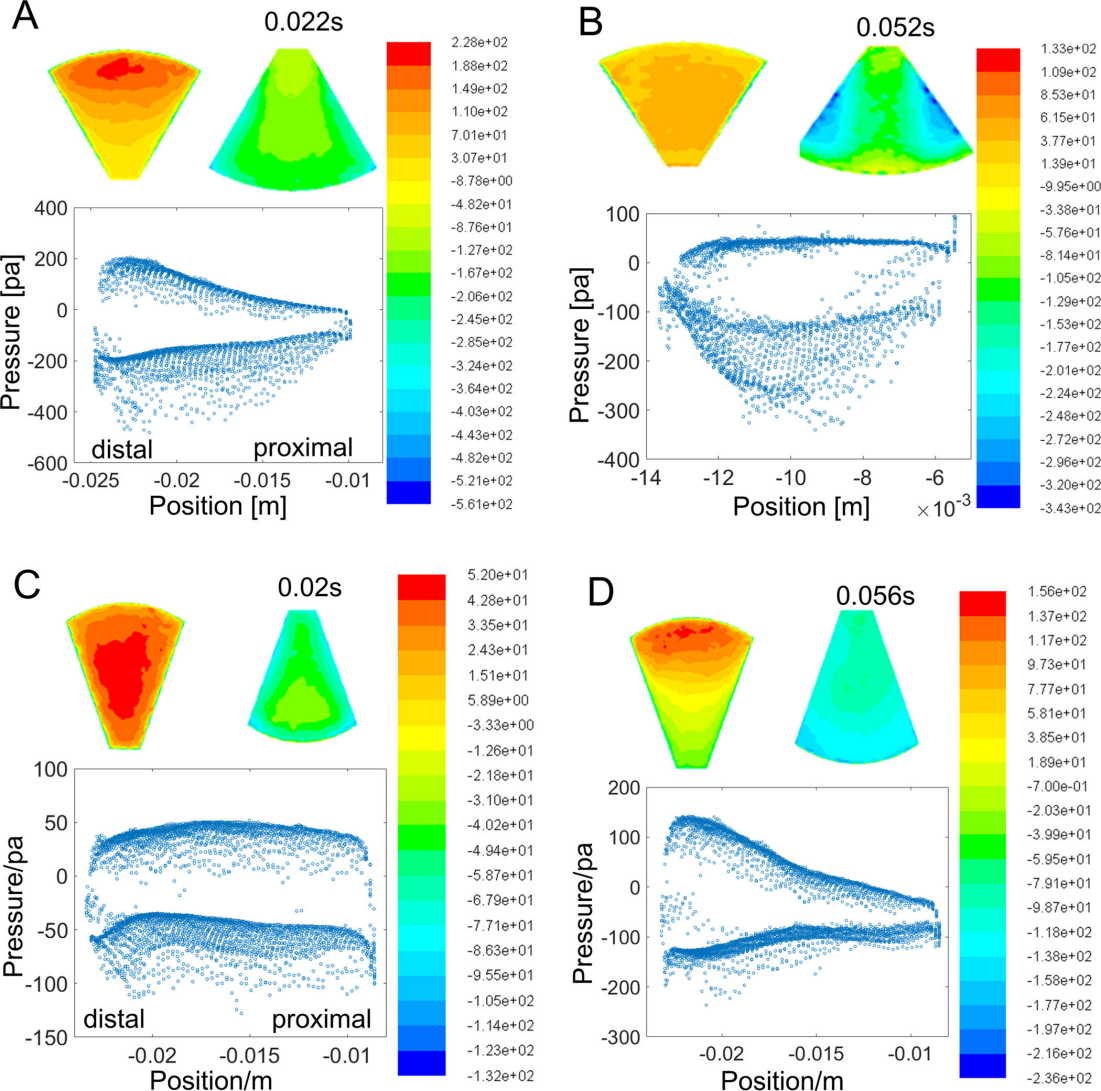
**Pressure distributions of the frog webbed feet.** Colors on the foot surfaces indicate the pressure magnitudes. The bottom faces repel water away, and pressure was positive. While the back faces absorb water, and the pressure was negative. The pressure values on the foot grid from the proximal to the distal end of the foot are drawn as blue open circles. (A) The results from the aquatic frog's swimming trial at the time of 0.022 s; (B) the results from the aquatic frog's swimming trial at the time of 0.052 s; (C) the results from the terrestrial frog's swimming trial at the time of 0.02 s; (D) the results from the terrestrial frog's swimming trial at the time of 0.056 s.

In the early propulsion (0.022 s), velocity on *Xenopus laevis*'s foot is distributed increasingly from the proximal to the distal due to the foot rotation, resulting in the pressures on both foot faces being linearly distributed (Fig. 4A). The similar pressure distributions were found on *Rana nigromaculata*'s foot (Fig. 4C), though the pressures concentrated as lines parallel to the axis due to the rare foot rotation in the early propulsion (0.02 s). In the late propulsion (0.056 s), *Rana nigromaculata*'s foot rotated, but the pressures still increased linearly from the proximal to the distal due to the foot rotation, as in Fig. 4D. The pressure distributions in the above situations showed regular relation with foot motion, so the thrust calculation could be solved by blade element approach based on the drag coefficient and kinematics. However, in the late propulsion (0.052 s) of *Xenopus laevis* (Fig. 4B), pressure distribution came to little relation with the foot motion which was still characterized by rotation. The bottom face had uniform pressures with small magnitudes as shown in Fig. 4B. The back face turned to be an irregular pressure distribution, and two low pressure centers on the sides were generated.

## DISCUSSION

### Frog swimming efficiency slightly exceeded drag-based swimming efficiency

In this study we used a model similar to Richards' research (Richards, 2008), where the frog model was built as planar linkages with webbed feet, but we further established the dynamic equations and computed the joint input work and propulsive efficiency according to the virtual work principle. The propulsive efficiency is the ratio between output and input work. In steady swimming, output work could be substituted by the drag work (mainly from the drag force on the body), but it may lead to underestimation of unsteady propulsion. Therefore, we computed the efficiency from the thrust and unsteady velocity so the propulsive efficiency results of velocity varied swimming could be obtained.

Fish studied the mechanical efficiencies of mammalian swimming with drag-based and lift-based propulsions (Fish, 1996), finding that propulsive efficiency is over 80% for some lift-based swimming while, at most, 33% for paddling or drag-based swimming. In his research, the swimming motions from terrestrial to aquatic mammals are paddling, rowing and oscillation. However, the longer limbs and webbed feet make frog swimming motion distinguishable from the above, and the propulsive efficiency of the aquatic frog exceeded that of common drag-based propulsion. Foot rotation constitutes the main motion during aquatic swimming, analogous to lift-based swimming mammals utilizing fin oscillation. This suggests that a lift mechanism might contribute to the thrust. However, a large gap remains between the lift-based swimming pattern of the aquatic frog and those utilized by many other aquatic animals. This highlights the necessity for hydrodynamic analysis for the raised efficiency.

### Lift mechanism improves the swimming efficiency

The propulsive mechanism of frog swimming was researched via theoretical fluid mechanics (Richards, 2008) or experimental methods (Nauwelaerts et al., 2005; Stamhuis and Nauwelaerts, 2005; Johansson and Lauder, 2004) to compute the thrust during propulsion, but only a few studies researched the propulsive efficiency. Frog swimming kinematics of diverse patterns were studied, but few have concluded that a lift mechanism was involved in swimming. Therefore, the exact relationship between swimming efficiency and kinematics in frogs remained unclear. To make a contribution to the research in frogs, the propulsive efficiency in some swimming trials was analyzed from CFD results. From the results in Fig. 3, the terrestrial frog showed no lift feature, while the aquatic frog generated a lift thrust in the second half of the propulsion. Drag-based thrust is generated from the foot backward movement relative to swimming direction. Therefore, as the frog moves forward, the foot backward movement will decrease due to adding the body velocity and the foot is eventually unable to generate drag-based thrust, but lift-based thrust continues to work through rotation. That is considered as the reason of the higher efficiency in cases 3 and 4.

The functions of the rotational movement in the two swimming patterns differs by the rotation stroke. During the two rotation patterns, the foot angle showed apparently different patterns. For the aquatic, the foot rotated intensely at the beginning and kept rotating, resulting in a lift-involved thrust; while for the terrestrial, the foot rotated slowly at the beginning when it remained at about 90° to the swimming axis, so the rotation was utilized to add the moving component along the swimming axis. These kinematic characteristics explained the drag thrust as in the previous study (Richards, 2008). However, from the CFD results, aquatic swimming pattern generated a different pressure distribution with the U-shaped vortex ring (Fig. 3B). In the late propulsion (0.052 s) of *Xenopus laevis*, the back face turned to be an irregular pressure distribution with two low pressure centers on the sides, which is supposed to be due to the lift mechanism. The negative pressure on back face was larger than that on bottom face, but both faces contribute to swimming impulse (Fig. 4B). However, in the late propulsion (0.052 s) of *Rana nigromaculata*, pressure on the bottom face still generated thrust as in the early propulsion, but the pressure on the back face dropped from positive to negative pressure due to the foot forward moving with body as in Fig. 4D. Therefore, the back faces contribute more to the swimming impulse and aquatic swimming seemed to be more efficient.

Johansson and Lauder conducted a series DPIV experiments to research the frog propulsion mechanism (Johansson and Lauder, 2004). Johansson analyzed the flow structure results and found that the vortex rings shedding from the terrestrial frog, *Rana pipiens*, was different from the lift-dominant mechanism and hadn't computed the propulsive efficiency in the frog. The frog in their research is similar to *Rana nigromaculata* in this paper, both known as terrestrial frogs. The terrestrial results from cases 1 and 2 were consistent with theirs; however, the lift mechanism sign (Johansson and Norberg, 2003) in their exploration was detected in cases 3 and 4. From the propulsive efficiency contrast, lift-involved mechanism proved to improve the swimming efficiency.

### Swimming patterns and foot shapes in frog swimming

Based on the hydrodynamic analysis from the four cases, swimming pattern is a main factor for propulsive efficiency, and webbed-foot shape and size also alter the performance and efficiency.

Swimming pattern comparisons were also conducted by Richards (Richards, 2010), but by the theoretical fluid mechanics, thus receiving lack of pressure distribution and flow structure data to reveal the lift-involved thrust. In his research, propulsive efficiency was not computed, but the impulses the webbed foot generated to propel frog swimming were derived. Our results agree with the kinematic results that the rotation in aquatic swimming is the main power source, while translational components contribute more in the terrestrial swimming. The diverse propulsive strategies from terrestrial to aquatic frog found in their research suggest the utilization ability of lift thrust which is considered to be a key factor for the higher propulsive efficiency in cases 3 and 4. Therefore, more works should be done to explore the vortex-based efficiency in this study.

The foot variations not only caused the shifts in swimming performance and propulsive efficiency, as previously explained, but also influenced the thrust and input work (Richards and Clemente, 2012) that is the muscle contraction and force output which can be computed by Eqns 4 and 5. According to the thrust calculation method in Richards' research (Richards, 2008), the thrust force is calculated according to the foot points' velocities and accelerations which are preset as linearly distributed in the foot. In the four cases, the aquatic foot is shorter than the terrestrial foot, so the terrestrial foot should increase the velocities of the foot points and thus increase the thrust if the pressure distribution is also linear with the velocity distribution in the CFD simulation. This hypothesis is observed in cases 1 and 2 since the terrestrial foot displayed better performance and propulsive efficiency. However, the results in Fig. 4B display two apparent low pressure cores in the middle of the foot sides, making the pressure distribution nonlinear, and given the decreased propulsive efficiency from case 4 to case 3, the foot shape function in the lift-involved thrust may be different from in the drag-based thrust as in the terrestrial swimming. Therefore, the results suggest the swimming patterns play a more important role and lift-involved propulsion leads to the higher propulsive efficiency.

### Discussion on Nauwelaerts and Aerts (2003)’s hypothesis about the small impulse in swimming

In Nauwelaerts and Aerts (2003)'s study, it was stated that the impulses during swimming were a lot smaller than the ones during terrestrial locomotion. The interpretation was that a faster extension would lead to too much slip of the feet and therefore a smaller impulse. To investigate the hypothesis, pressure distributions of the webbed feet were extracted from a simulation as shown in Fig. 4. The distal part of the foot is faster due to the rotation. In the early propulsion (0.022 s in Fig. 4A) of *Xenopus laevis* and late propulsion (0.056 s in Fig. 4D) of *Rana nigromaculata*, distal parts have larger velocity than the proximal, consistent with the pressure distribution. Therefore, the high speed of the foot resulted in a high pressure distribution, so impulses during the above situations are not limited by the fast extension. The thrust during swimming is generated by the relative speed and acceleration of the foot, and the body's movement will compromise the effective speed of the foot; whilst in jumping there is a solid ground to act on and gravity needs more power to overcome, resulting in a larger impulse in jumping than in swimming. Other research also handled this problem (Richards and Clemente, 2013), and concluded the muscle's property and hydrodynamic characteristics are tuned to make a reasonable extension in frog swimming with muscle contracting at 1/3 of the maximum shortening velocity. The differences between swimming and jumping impulses lie in many factors including environment and muscular system, but a faster extension would benefit swimming impulse though it may be still smaller than that in jumping.

### Conclusion

This paper focused on terrestrial swimming and aquatic swimming patterns in conjunction with various webbed feet. The frog feet were modeled to represent different foot shapes and sizes, while the frog hind legs were unified as a two-link mechanism of the same sizes in order to calculate propulsive efficiency. Based on the CFD simulation results, we can conclude that propulsive efficiency in aquatic frogs (43.11%) exceeds that in drag-based animals (33% maximum), but it is still far from the efficiency of lift-based propulsion. Meanwhile, propulsive efficiency in terrestrial frog (29.58%) fell within the range of drag-based propulsion. The foot makes a difference in swimming performance and efficiency, but the main factor is the swimming pattern.

## MATERIALS AND METHODS

### Animal model

To represent the typical terrestrial and aquatic frog species, the frog model and kinematic data were based on *Rana nigromaculata* [5.37±0.61 g (mean±s.d.) body mass; 36.73±2.27 mm snout-vent length; *N*=3 frogs] and *Xenopus laevis* [4.77±0.32 g (mean±s.d.) body mass; 32.53±2.08 mm snout-vent length; *N*=3 frogs]. To obtain the kinematic data for frog swimming, the observation experiments were conducted (Zhang et al., 2014). Animals were housed and taken good care of in aquaria at room temperature, fed with tubificidae. No surgery was conducted and the experiments were conducted in compliance with local ethical regulations on animal experiments. During experiments, the frogs could swim freely in aquaria and were filmed with a high speed camera at 400 frame/s. The camera was positioned in front of the aquarium to obtain a lateral view of the frog, and a mirror was set at 45° in the aquarium to get the top view. The mass and size information were measured immediately after the observation experiments and processed in the software MATLAB (The MathWorks, Natick, MA, USA).

**Fig. 5.**
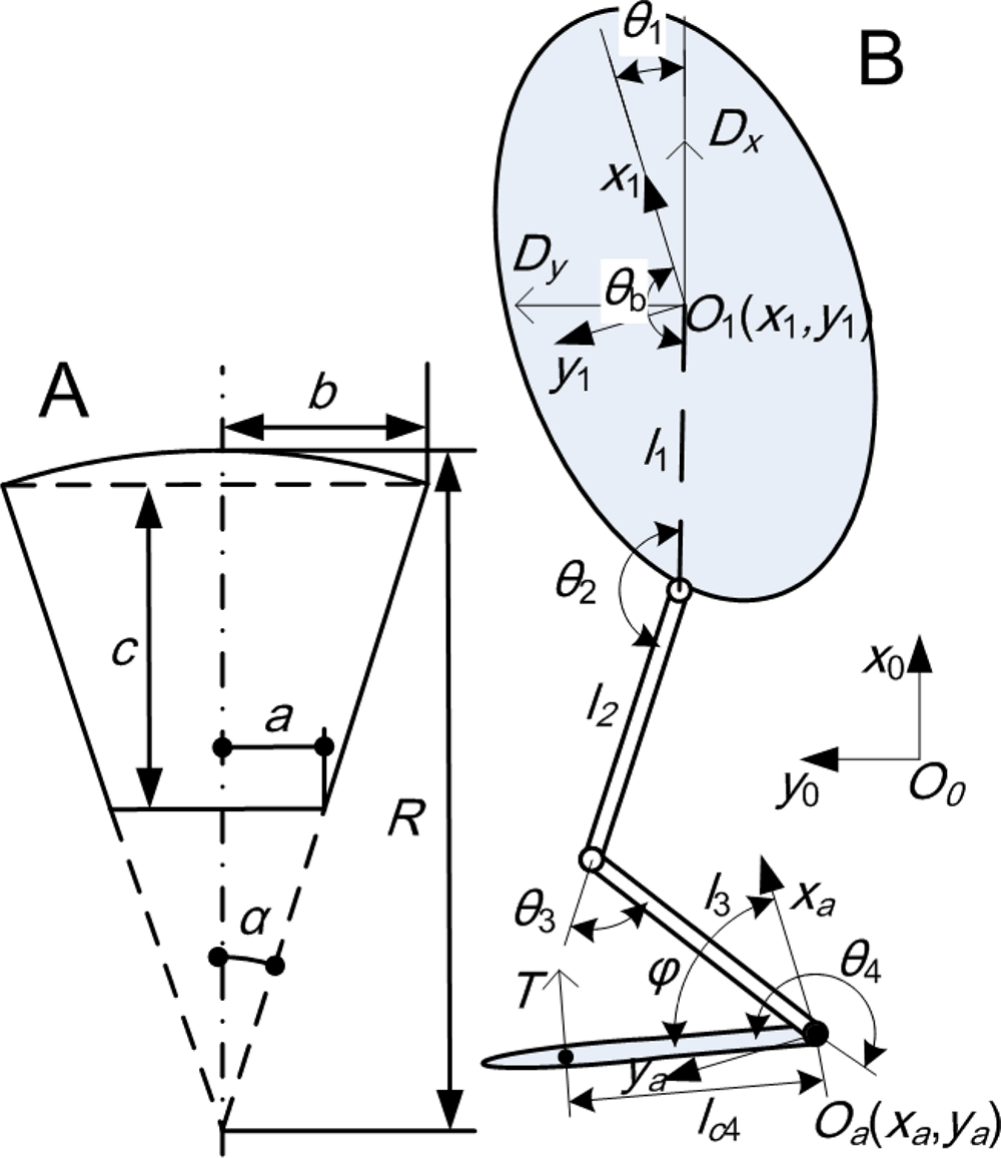
**Frog model.** (A) The parameters of the webbed foot; (B) the leg length and coordinate system establishment and forces exerted on frog.

Previous research has shown that the frog webbed foot's proximal end trajectory relative to the body traces variant lines including straight and elliptic lines, accelerating or decelerating along the track. Meanwhile, the foot also rotates about the ankle. The swimming patterns can be divided into aquatic swimming pattern and terrestrial swimming pattern, which are distinguished by ankle's moving tracks and the delays between foot's translational and rotational acceleration peaks (Zhang et al., 2015). Therefore, swimming patterns based on foot motions are an effective indicator to characterize a frog's moving patterns. Although a frog has multiple degrees of freedom (DOF) during swimming, the planar dimensions of the frog are sufficient to establish a model according to swimming observations and morphological measurements. The frog model can be simplified as shown in Fig. 5 (Fan et al., 2015), and the system can be described by DOFs of foot and body positions stated as {*x*_a_, *y*_a_, *φ*, *x*_1_, *y*_1_, *θ*_1_} to describe the motion in CFD simulations.

The webbed foot model in Fig. 5A mimics shapes found in frogs (Goldberg and Fabrezi, 2008), which vary but can be abstracted as a delta shape with one round edge. In the whole swimming process, the foot has to fully open in propulsion and close when hind limbs are folded. The open to close deforming of the foot is difficult to model in our present simulation. Therefore, only the propulsive phase is simulated when the foot can be treated as a rigid plate. The foot can be described according to variable aspect ratios (AR=fin span^2^/fin area=4*b*^2^/*area*^2^) and areas (Richards and Clemente, 2012).

The foot motion is driven by the hind legs, which are different among frogs in terms of leg length ratio and joint rotations. In order to establish an equivalent contrast, the frog's hind leg system was simplified as a two-link mechanism including the linkages of thigh and calf, and joints of hip, knee and ankle, as shown in Fig. 5B. The origin of the coordinate frame {*O*_1_} fixed with the body is denoted as (*x*_1_, *y*_1_) in the world coordinate frame {*O*_0_}. The coordinate frame fixed with the ankle is marked as {*O_a_*}. The DOFs in joint space can be expressed as {*θ*_1_, *θ*_2_, *θ*_3_, *θ*_4_, *x*_1_, *y*_1_} to calculate the dynamics. The body frame and world frame overlap at the beginning, and the hip is located at (*l*_1_cos*θ*_b_, *l*_1_sin*θ*_b_) in the body frame. The hip location was determined by the body structure, making it a constant.

### Dynamic equations

Joint torques in the two-link mechanism can be computed based on the hydrodynamic results and frog dynamic equations, after which swimming efficiency in the propulsive phase can be obtained. This study used the Langrange form dynamic equation as follows:
(1)
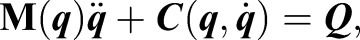
where **M** is 6×6 mass matrix, which is symmetric and nonsingular; ***C*** denotes the 6×1 vector that implicitly includes centrifugal, Coriolis, and gravity terms; ***q*** is the 6×1 vector of the system's general DOF; and ***Q*** represents the 6×1 vector of general forces or torques.

According to the virtual work principle (Zhang and Song, 1993), elementary work *δW* can be calculated according to the force analysis of the frog model as shown in Eqn (2):
(2)
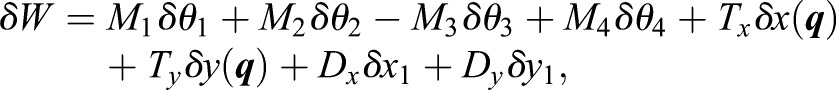
where *M*_1_ is the moment caused by hydrodynamic forces on the body, and in the straight swimming, *M*_1_=0 for the symmetrical forces on the body as it swims in a straight line. Similarly, *M*_2_, *M*_3_ and *M*_4_ represent the joint driving torques at the hip, knee, and ankle, respectively; *D_x_* and *D_y_* are the hydrodynamic forces on the body; *T_x_* and *T_y_* are the hydrodynamic forces on the webbed foot; and *δx* and *δy* are the elementary displacement of the forces *T_x_* and *T_y_*. Elementary work *δW’* can also be expressed by Eqn (3):
(3)
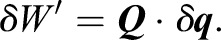
After equalizing Eqns (2) and (3), the joint driving torques can be computed by Eqn (4) as follows:
(4)
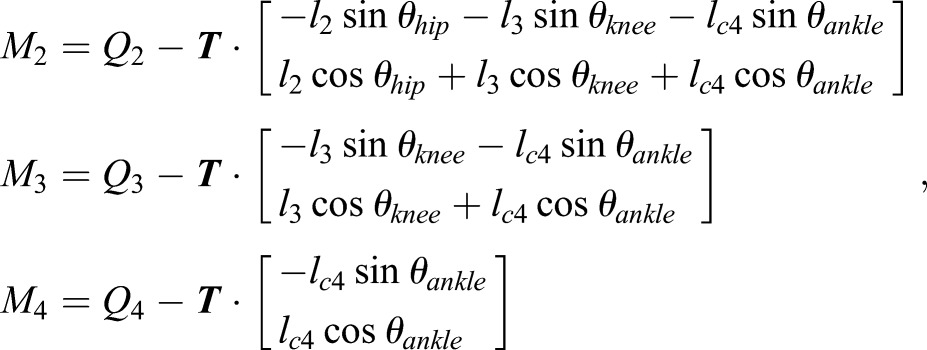
where *θ*_hip_, *θ*_knee_, and *θ*_ankle_ denote the angles of the joints in the world coordinate frame as: *θ*_hip_=*θ*_1_+*θ*_2_+*θ*_b_, *θ*_knee_=*θ*_1_+*θ*_2_+*θ*_3_+*θ*_b_, *θ*_ankle_=*θ*_1_+*θ*_2_+*θ*_3_+*θ*_4_+*θ*_b_.

Propulsive efficiency is the ratio of thrust power work to the total mechanical work produced, and it can be solved by Eqn (5) (Fish, 1994):
(5)
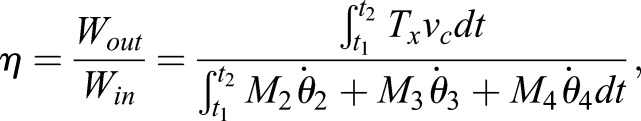
where *η* is the average efficiency during the propulsive phase; *W*_out_ is the output work from thrust power; *W*_in_ is the total mechanical work produced; *t*_1_ and *t*_2_ are the beginning and end times, respectively, of the propulsive phase; and *v*_c_ is the velocity of the center of mass.

### CFD simulations

**Fig. 6.**
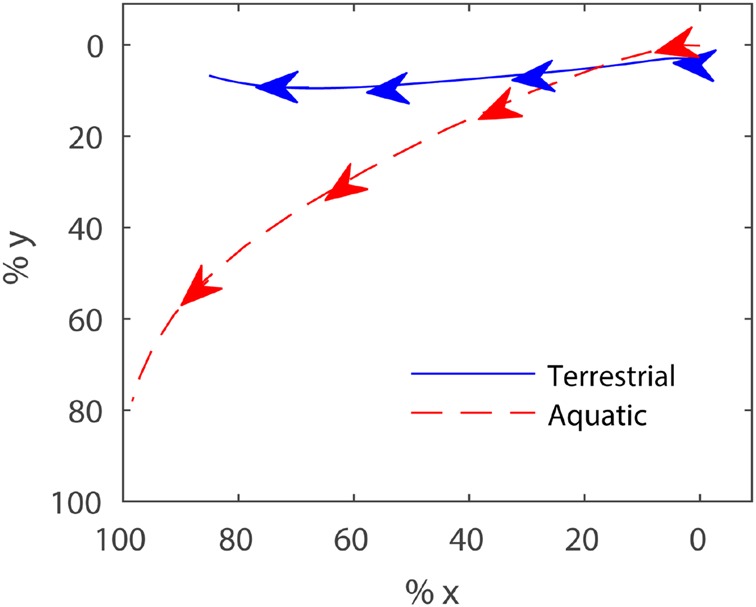
**Ankle trajectories.** The blue solid line shows the percent track from the propulsive phase of a terrestrial frog's swimming trial and the red dashed line shows the data from an aquatic frog. The ankles move in the direction of the arrows.

**Fig. 7.**
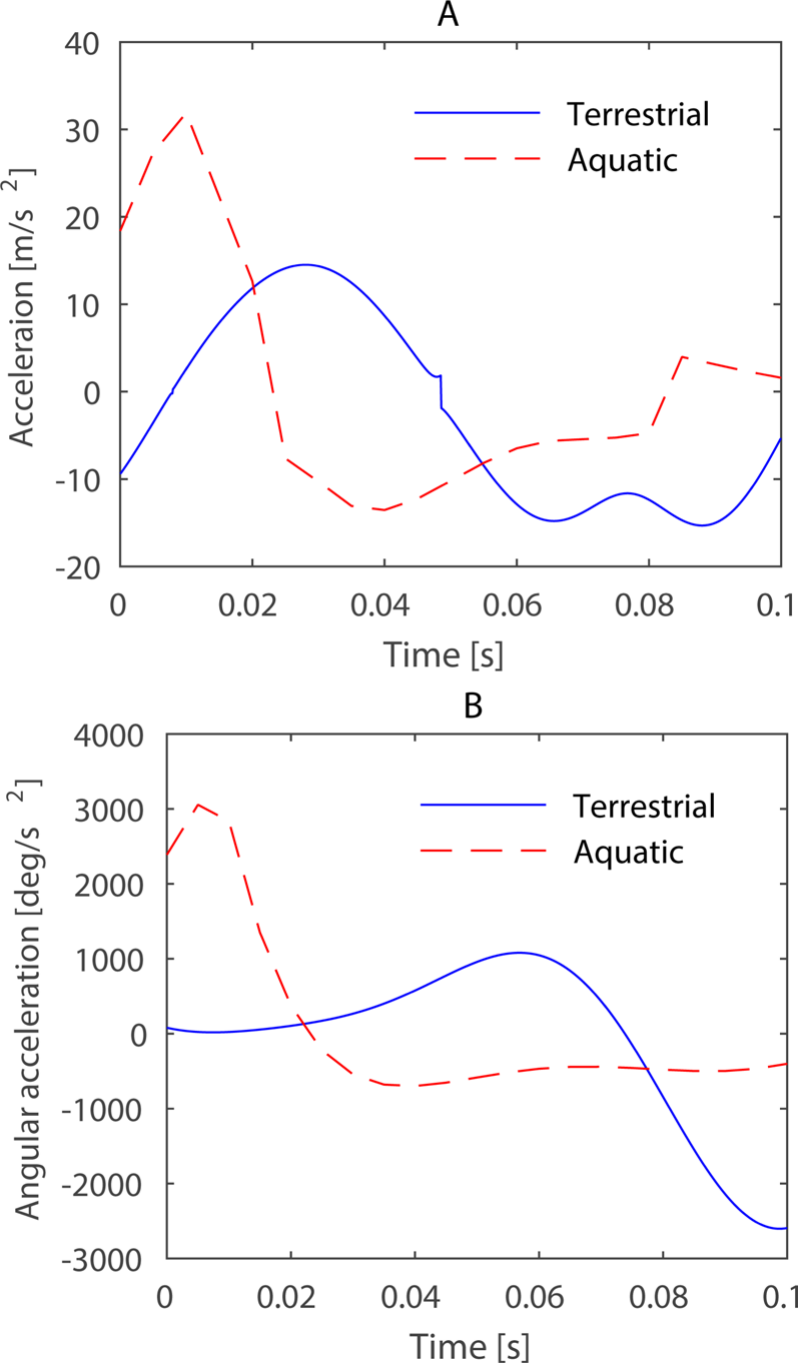
**Ankle accelerations along their tracks.** (A) The acceleration along the ankle tracks in Fig. 6. (B) The angular acceleration of the foot which rotates about the ankle. The blue solid line represents the terrestrial frog and the red dashed line the aquatic frog.

From six swimming trials of *Xenopus laevis* and five trials of *Rana nigromaculata*, one representative swimming trial of each frog was chosen for simulation taking into account that the aquatic frog has a more remarkable lateral and rotational movement than the terrestrial frog. In *Xenopus laevis*'s trial, it swam with the maximum velocity of 0.51 m/s and the propulsive phase is about 0.065 s, while in *Rana nigromaculata* the maximum velocity is 0.44 m/s and the propulsive phase is about 0.072 s, so the close performances made them appropriate representatives. Fig. 6 shows the ankle track data of the two swimming trials. Displacement along the x-axis for *Rana nigromaculata*, a terrestrial frog, reached 22.3 mm, with little movement observed along the y-axis; in contrast, the x-axis movement of *Xenopus laevis*, an aquatic frog, reached 9.7 mm that was as almost much as that along the y-axis. Fig. 7A shows the accelerations in the ankle trajectories. The acceleration patterns were consistent across both aquatic and terrestrial frogs. The ankle experienced a marked acceleration peak during the first stage of the propulsive phase, then slowly decelerated in the long second stage. The patterns of acceleration magnitude were similar, but the diversity of ankle trajectories may create different hydrodynamic effects. Fig. 7B illustrates the webbed foot's angular acceleration around the ankle's z axis. The patterns of angular acceleration proved to be opposites between the two groups. The terrestrial frog slowly rotated its foot at first and then increased angular acceleration, such that the foot remained perpendicular to the swimming axis while the ankle experienced intense backward acceleration and velocity. This pattern of motion maintained a large surface area during drag-based thrust generation. For the aquatic swimming pattern, the peaks of the ankle's acceleration and angular acceleration were almost simultaneous, with deceleration occurring in the rest phase; this pattern created rotationally predominant swimming. These data were used to present the two different swimming patterns.

The webbed foot models for terrestrial and aquatic frogs were built into the CFD simulations, and the kinematic data were input to the simulation to realize the two swimming patterns. The parameters of aquatic frog models are as follows: *l*_1_=*l*_2_=*l*_3_=14 mm, *θ*_b_=120deg *a*=1.5 mm, *R*=17.5 mm, *α*=30°, AR=1.97, area=155.7 mm^2^, which will be used in the following simulation cases 2 and 4. To maintain the same area, the terrestrial frog foot was designed with the same AR of the observed frog but the larger area of 155.6 mm^2^. The parameters for the lengthened terrestrial foot are as follows: *l*_1_=*l*_2_=*l*_3_=11 mm, *θ*_b_ =120deg, *a*=1.5 mm, *R*=22.8 mm, *α*=18°, AR=1.28, area=155.6 mm^2^, which will be used in the following simulation cases 1 and 3. While the parameters for original terrestrial frog foot are as follows: *a*=1.5 mm, *R*=19.6 mm, *α*=18°, AR=1.27, area=113.2 mm^2^. Analysis of swimming performance based on both swimming patterns and foot structures proceeded in CFD simulations according to the four cases below.

Case 1: swimming using a terrestrial swimming pattern with lengthened terrestrial foot models.

Case 2: swimming using a terrestrial swimming pattern with aquatic foot models.

Case 3: swimming using an aquatic swimming pattern with lengthened terrestrial foot models.

Case 4: swimming using an aquatic swimming pattern with aquatic foot models.

This paper utilized a CFD-based self-propulsion simulation in the software of FLUENT (ANSYS, PIT, PA, USA) to compute the hydrodynamic forces on the webbed foot. In the CFD simulation, the frog body and limbs were set in a fluid field as inner boundary walls that could be moved by redrawing the calculating mesh. The hydrodynamic forces acting on the walls were considered to be external forces on the frog. With the solid dynamic model of the frog embedded in the CFD process, the wall mesh could be redrawn. The self-propulsion simulation could calculate the interactions of the solid dynamics and hydrodynamics through the CFD simulation with trajectories based on data from observations and trajectory planning, resulting in a natural body movement. The self-propulsion simulation ensures that the body's motion is derived from mutual reactions between fluid and the frog, thus guaranteeing reliable results for use in dynamic analysis. The details about the simulation is in Fan et al. (2014).

## References

[BIO022913C1] Chu W.-S., Lee K.-T., Song S.-H., Han M.-W., Lee J.-Y., Kim H.-S., Kim M.-S., Park Y.-J., Cho K.-J. and Ahn S.-H. (2012). Review of biomimetic underwater robots using smart actuators. *Int. J. Prec. Eng. Manuf.* 13, 1281-1292. 10.1007/s12541-012-0171-7

[BIO022913C2] Fan J., Zhang W., Zhu Y. and Zhao J. (2014). CFD-based self-propulsion simulation for frog swimming. *J. Mech. Med. Biol.* 14, 1440012 10.1142/S0219519414400120

[BIO022913C3] Fan J., Qiu Y., Zhang W. and Wang H. (2015). Mechanical design of frog inspired swimming robot (in Chinese). *Robot* 37, 168-175. 10.13973/j.cnki.robot.2015.0168

[BIO022913C4] Fish F. E. (1994). Influence of hydrodynamic design and propulsive mode on mammalian swimming energetics. *Aust. J. Zool.* 42, 79-101. 10.1071/ZO9940079

[BIO022913C5] Fish F. E. (1996). Transitions from Drag-based to Lift-based propulsion in mammalian swimming. *Amer. Zool.* 36, 628-641. 10.1093/icb/36.6.628

[BIO022913C6] Goldberg J. and Fabrezi M. (2008). Development and variation of the anuran webbed feet (Amphibia, Anura). *Zool. J. Linn. Soc.* 152, 39-58. 10.1111/j.1096-3642.2007.00345.x

[BIO022913C7] Johansson, L. C. and Lauder G. V. (2004). Hydrodynamics of surface swimming in leopard frogs (Rana pipiens). *J. Exp. Biol.* 207, 3945-3958. 10.1242/jeb.01258 15472025

[BIO022913C8] Johansson L. C. and Norberg R. A. (2003). Delta-wing function of webbed feet gives hydrodynamic lift for swimming propulsion in birds. *Nature* 424, 65-68. 10.1038/nature01695 12840759

[BIO022913C9] Kim H.-J., Song S.-H. and Ahn S.-H. (2013). A turtle-like swimming robot using a smart soft composite (SSC) structure. *Smart Mater. Struct.* 22, 014007 10.1088/0964-1726/22/1/014007

[BIO022913C10] Nauwelaerts S., Stamhuis E. J. and Aerts P. (2005). Propulsive force calculations in swimming frogs I. A momentum–impulse approach. *J. Exp. Biol.* 208, 1435-1443. 10.1242/jeb.01509 15802667

[BIO022913C18] Nir S., Ruchaevski I., Shraga S., Shteinberg T. and Ben Moshe B. (2012). A jellyfish-like robot for mimicking jet propulsion. *2012 IEEE 27th Convention of Electrical and Electronics Engineers November 14–17*, pp.1-5, Eilat, Israel.

[BIO022913C11] Rahman M. M., Sugimori S., Miki H., Yamamoto R., Sanada Y. and Toda Y. (2013). Braking performance of a biomimetic squid-like underwater robot. *J. Bionic Eng.* 10, 265-273. 10.1016/S1672-6529(13)60222-X

[BIO022913C12] Richards C. T. (2008). The kinematic determinants of anuran swimming performance: an inverse and forward dynamics approach. *J. Exp. Biol.* 211, 3181-3194. 10.1242/jeb.019844 18805818

[BIO022913C13] Richards C. T. (2010). Kinematics and hydrodynamics analysis of swimming anurans reveals striking inter-specific differences in the mechanism for producing thrust. *J. Exp. Biol.* 213, 621-634. 10.1242/jeb.032631 20118313

[BIO022913C14] Richards C. T. and Clemente C. J. (2012). A bio-robotic platform for integrating internal and external mechanics during muscle-powered swimming. *Bioinspir. Biomim.* 7, 016010 10.1088/1748-3182/7/1/016010 22345392

[BIO022913C15] Richards C. T. and Clemente C. J. (2013). Built for rowing: frog muscle is tuned to limb morphology to power swimming. *J. R. Soc. Interface* 10, 20130236 10.1098/rsif.2013.0236 23676897 PMC3673160

[BIO022913C16] Roper D. T., Sharma S., Sutton R. and Culverhouse P. (2011). A review of developments towards biologically inspired propulsion systems for autonomous underwater vehicles. *Procee. Inst. Mech. Eng. M J. Eng. Maritime Env.* 225, 77-96. 10.1177/1475090210397438

[BIO022913C17] Sfak I., Lane D. and Davies J. (1999). Review of fish swimming modes for aquatic locomotion. *IEEE J. Ocean. Eng.* 24, 237-252. 10.1109/48.757275

[BIO022913C19] Stamhuis E. J. and Nauwelaerts S. (2005). Propulsive force calculations in swimming frogs. II. A vortex ring approach. *J. Exp. Biol.* 208, 1445-1451. 10.1242/jeb.01530 15802668

[BIO022913C20] Zhang C.-D. and Song S.-M. (1993). An efficient method for inverse dynamics of manipulators based on the virtual work principle. *J. Robot. Syst.* 10, 605-627. 10.1002/rob.4620100505

[BIO022913C21] Zhang W., Fan J., Zhu Y., Qiu Y. and Zhao J. (2014). A method for mechanism analysis of frog swimming based on motion observation experiments. *Adv. Mech. Eng.* 6, 403057 10.1155/2014/403057

[BIO022913C22] Zhang W., Liu G., Fan J. and Cai H. (2015). Foot trajectory planning of frog swimming based on propulsion mechanism. *Proceedings of the 2015 IEEE Conference on Robotics and Biomimetics December 6-9*, pp. 929-933, Zhuhai, China.

